# TRPV4 activates the Cdc42/N-wasp pathway to promote glioblastoma invasion by altering cellular protrusions

**DOI:** 10.1038/s41598-020-70822-4

**Published:** 2020-08-25

**Authors:** Wei Yang, Peng-fei Wu, Jian-xing Ma, Mao-jun Liao, Lun-shan Xu, Liang Yi

**Affiliations:** 1 Department of Neurosurgery, Xinqiao Hospital, Army Medical University (Third Military Medical University), Chongqing, 400037 P.R. China; 2 Department of Neurosurgery, Daping Hospital and Institute Research of Surgery, Army Medical University, 10# Changjiangzhi Road, Daping, Yuzhong District, Chongqing, 400042 China

**Keywords:** Cancer, Cell biology, Drug discovery, Molecular biology

## Abstract

The invasion ability of glioblastoma (GBM) causes tumor cells to infiltrate the surrounding brain parenchyma and leads to poor outcomes. Transient receptor potential vanilloid 4 (TRPV4) exhibits a remarkable role in cancer cell motility, but the contribution of TRPV4 to glioblastoma metastasis is not fully understood. Here, we reported that TRPV4 expression was significantly elevated in malignant glioma compared to normal brain and low-grade glioma, and TRPV4 expression was negatively correlated with the prognosis of glioma patients. Functionally, stimulation of TRPV4 promoted glioblastoma cell migration and invasion, and repression of TRPV4 hindered the migration and invasion of glioblastoma cells in vitro. Molecularly, TRPV4 strongly colocalized and interacted with skeletal protein-F-actin at cellular protrusions, and TRPV4 regulated the formation of invadopodia and filopodia in glioblastoma cells. Furthermore, the Cdc42/N-wasp axis mediated the effect of TRPV4-regulated cellular protrusions and invasion. Foremost, TRPV4 inhibitor treatment or downregulation of TRPV4 significantly reduced the invasion-growth of subcutaneously and intracranially transplanted glioblastoma in mice. In conclusion, the TRPV4/Cdc42/wasp signaling axis regulates cellular protrusion formation in glioblastoma cells and influences the invasion-growth phenotype of glioblastoma in vivo. TRPV4 may serve as a prognostic factor and specific therapeutic target for GBM patients.

## Introduction

Glioblastoma (GBM) is the most common primary malignant tumor in the central nervous system (CNS). The current treatment protocol for glioblastoma patients is surgery and radiation therapy followed by treatment with temozolomide. Despite the development of multimodal treatment, the median life expectancy for patients with GBM is still approximately 14 months, and fewer than 5% of patients survive beyond 5 years of diagnosis^1^. Unlike other malignant solid cancers, glioblastoma cells prefer to invade diffusely rather than create systemic metastases. The aggressive feature of glioblastoma cells is their invasion of surrounding normal brain tissues, which is the primary reason for incomplete surgical resection, chemo-radiation resistance and inevitable recurrence. There are very limited possibilities to target these processes therapeutically^2^. In light of the poor outcome from current therapies in glioblastoma patients, a better understanding of GBM invasion may provide more effective interventions to manage this devastating disease.

In particular, GBM cells always migrate along the basement membrane of blood vessels intraparenchymally or follow white matter tracts^3,4^. In this process, GBM cells must adjust their morphology to interact with the surrounding environment to infiltrate the surrounding parenchyma^5^. Because the extracellular space in which glioblastoma cells move is much smaller than that for migrating cells^6^, migrating glioblastoma cells must remodel their cytoskeleton to change cell morphology to permeate this narrow space^7^. Meanwhile, GBM cells form cellular protrusions at the leading edge to degrade extracellular matrix (ECM) and move the cell body forward^8^. The protrusive structures are termed focal adhesions, filopodia, lamellipodia and invadopodia in tumors based on their morphological, structural and functional characteristics^9^. In general, invadopodia and filopodia serve as sensors of the external microenvironment and as plasma membrane extensions that form initial contacts with the ECM. Therefore, invadopodia and filopodia are considered essential processes in the migration and invasion of glioblastoma cells^10^. They also allow cancer cells to coordinate ECM degradation with cell motility and to facilitate cell migration^11^. Cdc42, which belongs to the small Rho-family GTPase, is a key regulator of actin cytoskeletal arrangement and has an essential role in the glioma cell migration process^12^. Cdc42 can directly bind to several actin-regulatory proteins, such as WASP, mDIA and WAVE. These cascades directly regulate the behavior and organization of the actin cytoskeleton. A recent study has shown that the Cdc42 target downstream regulator of actin influences cell invasion and filopodia formation^13^.

Transient receptor potential vanilloid type 4 (TRPV4) ion channels are Ca^2+^-permeable nonselective cation channels that belong to the transient receptor potential vanilloid subfamily. This receptor exhibits a remarkable range of expression throughout the human body^14^. Previous research has demonstrated its functional relevance to the regulation of body osmolarity, mechanosensation, temperature sensing and vascular regulation^15^. Moreover, a recent study implied that TRPV4 drives breast cancer cell and breast tumor-derived endothelial cell migration^16–18^. Whether TRPV4 contributes to the biology of glioblastoma is undiscovered. In this study, we evaluated the expression of TRPV4 in GBM and revealed its role as a tumor promoter, which role is mediated by Cdc42/N-wasp activation and promotion of cellular protrusion formation.

## Materials and methods

### Human tissue specimens, cell lines and culture

Ten normal brain tissus, fifteen low grades glioma and fifteen high grades glioma tissues were obtained from patients who are undergoing surgery at the Department of Neurosurgery, Daping Hospital of Army Medical University. Informed consent was obtained from all patients and the study was approved by the Ethics Committee of the Daping Hospital of Army Medical University. All relative experiments were performed in accordance with the relevant guidelines. and regulations. These glioma tissues were graded by at least 2 experienced clinical pathologists according to the WHO classification system. Glioblastoma cell lines: Rat (C6), Human (U87, A172 and U251), Mouse (GL261) and Human embryonic kidney cell line (HEK293T) were purchased from American type culture collection. STR confirmation for U87 and U251 cell line was conducted by the Institute Research of Surgery of Army Medical University. All cell lines were cultured in DMEM/F12 containing 10% fetal bovine serum, penicillin (107 U/L) and streptomycin (10 mg/L) at 37 °C in a humidified incubator with 5% CO_2_.

### Reagents and antibodies

GSK1016790A, GSK2193874, DMSO, and DAPI were obtained from Sigma Aldrich (USA), Wiskostatin was purchased from Santa-cruz (USA), ML141 was purchased from MedChemExpress (China), Lipofectamine2000 was obtained from Invitrogen, FITC-phalloidin was purchased from Life Technological (USA), Rabbit anti-TRPV4 monoclonal antibody was purchased from Abcam (USA). Mouse Anti-F-actin monoclonal antibody was purchased from Beyotime (China).

### Analysis of patient data

The data was downloaded from TCGA database on April 3, 2018 (https://portal.gdc.cancer.gov). After deleted the uncertainty data and lacking of clinical data, the RNA-seq data contained 694 glioma patients information (527 low grade gioma and 167 GBM). Kaplan–Meier analysis was conducted with the TCGA-glioma, TCGA-gliomblastoma and TCGA-low grade glioma datasets. we used cutoff value at 85.5 to divide 694 glioma patients and cutoff value at 115 to divide 167glioblastoma patients into two groups (high expression and low expression) for analyze overall survival (OS) rates. we used cutoff value at 103.5 to divide 628 glioma patients and cutoff value at 45.5 to divide 161 glioblastoma patients into two groups (high expression and low expression) for analyze progression free survival (PFS) rates, we failed to found appropriate cutoff value to divide low grade glioma and make significantly difference between two groups. The OS and RFS curve and P value were analyzed by log-rank test.

### Lentivirus vector construction and infection

Two sequences of shRNA targeting human TRPV4 gene are GCCAGTGTATTCCTC.

GCTTTA and CGCTGCAAACACTACGTGGAA. We co-transfected Sh-TRPV4 vector or control vector (Genechem) with the packaging lentivirals (Genechem, China) into HEK293T cells using Lipofectamine 2000. Virus particles were harvested, concentrated and quantitated at 48 h after transfection. The U87 cells were infected with lentivirus particles or control lentivirus particles. Kept transfection MOI value at 1 according to GBM cell amount. 4 µg/ml Puromycin was added in U87-Ctr-SH cells and U87-TRPV4-SH cells at 72 h after infection for 3 days, then replaced with 0.5ug/ml Puromycin for 2 weeks to obtain the stable silence cells. lentivirus contain sh-Cdc42 was obtained from Genechem, the target sequences for Cdc42 gene are ACAGACAATTAAGTGTGTTGTTTAGTGAAGCCACAGATG.

TAAACAACACACTTAATTGTCTGC and ACAAGAGGATTATGACAGATTATA.

GTGAAGCCACAGATGTATAATCTGTCATAATCCTCTTGC. we transfected it into glioblastoma cell and selected stable U87-Cdc42-SH cell by Puromycin treated as above description.

### Cell migration and invasion assays

GBM Cells were seeded in 6-well plates and starved for 12 h after 90% confluence. A wounding line was scratched with 10 μL pipet tip, and added the serum-free medium to the plates. After 24 h of incubation. The migrated cells were monitored with Olympus inverted microscope and counted in 5 randomly selected fields to quantify the cell migration area with Image J software. Transwell filters (8.0 μM pore size, Corning, USA) were pre-coated with Matrigel (Corning, USA). 5 × 10^4^ cells in 100 μL of serum-free medium were seeded into the upper chamber after starved for 12 h. 600 µL of medium containing 5% fetal bovine serum was seeded into the lower chamber. Cells were incubated for 24 h and removed from the upper surface. Invaded cells on the lower surface were fixed with 4% paraformaldehyde, permeated with 0.3%TritonX-100, and stained with 5% crystal violet (Beyotime, China). Invaded cells were counted in 5 randomly selected fields with Iamge J software to quantify the invasive rate.

### Western blotting assay and co-Immunoprecipitation

Cells were washed in PBS and lysed in ice-cold RIPA buffer with PMSF and phosphatase inhibitor for the whole-cell protein. The equal amounts of proteins (30 µg/lane) were separated by SDS-PAGE and transferred onto PVDF membrane. The membrane was blocked and incubated with primary antibodies. Specific antibodies against TRPV4 (1:5,000), GAPDH(1:2,500), N-wasp (1:1,000) and p-N-wasp (1:1,000); The membrane was washed and incubated with secondary antibodies. Immunoblots were detected by Western-Bright Sirius HRP Substrate Kit (Advansta, USA) and exposed to ChemiDoc TMXRS + Image System (Bio-Rad, USA). The Image J was used to analyze the relative expression level of protein. For IP, cells were washed with ice-cold PBS and lysed in IP lysis buffer containing 1% protease inhibitor cocktail and phosphatase inhibitor. Lysates were clarified by centrifugation precleared by adding 1.0ug appropriate control IgG, together with 20 µL Protein A/G PLUS-Agarose (Santacruz, USA). Incubate at 4 °C for 30 min, centrifugated and transfered supernatant. Added 4 µL primary antibody and incubated for 1 h at 4 °C following 20 µL Protein A/G PLUS-Agarose. incubated at 4 °C on a rocker platform for overnight. Immunoprecipitates were washed three times with 1 mL of IP lysis buffer at 4 °C, and then boiled in loading buffer at 95 °C for 5 min. The beads were removed by centrifugation, and immunoprecipitated lysates were subjected to SDS-PAGE and western blotting analysis.

### Pull-down assays

Cells were chilled at 4  °C and lysed in an ice-cold RIPA buffer with 1 × PMSF for 10 min. The cell lysates were centrifugated at 12,000*g* at 4 °C for 10 min. the supernatant were quantified (30 µg/lane) and denatured with SDS-PAGE lysis buffer for measuring the total amount of the Cdc42 GTPases by Western blotting. For the pull-down assays, the supernatants were incubated with fusion protein between glutathione-S-transferase (GST) and the PBD domain of Cdc42-effector PAK (30 µg), linked to glutathione-Sepharose beads at 4 °C for 30 min. The beads were washed three times in lysis buffer and denaturated in SDS-PAGE lysis. The activated and total proteins were analyzed in parallel by Western blotting with rabbit monoclonal antibody against Cdc42 (1:2,500, CST, USA).

### Immunohistochemistry (IHC), immunofluorescence (IF) and actin staining

Human normal brain tissues and glioma tissues were fixed with 4% paraformaldehyde and cut into 4 μm sections. The slides were treated for antigen retrieval with sodium citrate for 20 min at 100  °C, incubated with primary and secondary antibodies, and enzyme conjugate horseradish peroxidase. The dilution of antibody: TRPV4 (1:100), Ki67 (1:100). Cells on cover slips were fixed at 72 h after shRNA transfection, or 24 h after GSK1 (10 nM) and GSK2 (100 nM) treated, blocked and permeated, then incubated with primary antibodies (TRPV4 1:100) and Fluorescence labeled second antibody (TRITIC-conjugated anti-rabbit). Actin was stained by FITC-phalloidin (1:200 dilution; Life, USA) at 4 °C overnight, The nucleus was stained with DAPI and examined with Zeiss LSM 780 Meta confocal microscope. The images were processed by ZEN software.

### Filopodia and invadopodia detection

FiloQuant installation in Fiji can be easily achieved through the ImageJ update site as previous described^19^. To analyze filopodia dynamics in the IF assays of U87 and GL261 cells, filopodia were first detected and analyzed using FiloQuant with the “single image FiloQuant” option, we used threshold value = 50 for cell edges detection, threshold value = 25 for Filopodia detection, Filopodia minimum size setting was 20 pixel. In addition, filopodia further than 30 pixels away from the detected cell edge were excluded using the “maximal distance from cell edges”option. Data was exported and analyzed by SPSS13.0 and Graphpad5.0. We calculated the number of invadopodia by 5 randomly select image of every group. The unbiased quantifications of invadopodia number were performed by two observers who were completely blinded to any group information. They counted the numbers of invadopodia from 5 randomly image of each group and calculated the data.

### Subcutaneous and orthotopic xenografts implantation and MRI scan

4-weeks old Immunodeficient athymic nude mice were purchased from the Experimental Animal Center of Army Medical University. Subcutaneous GBM transplantation model in nude mice was established by injection of 1 × 10^6^ U87-Ctr-SH and U87-TRPV4-SH cells which was diluted in 100 µL PBS respectively. Tumor value was calculated with formula Tv = length × width^2^/2 , tumor tissues were ground and extracted protein for western blotting. The orthotopic xenografts model in 4-weeks old nude mice was established by injection of 1 × 10^7^ U87 cells (diluted in 10 µL PBS) into the right striatum of mice by brain stereotaxic instrument (n = 16). The mice in the GSK2 group (n = 8) were intraperitoneally injected with GSK2 diluted in DMSO (20 nM/day/kg), the mice in control group (n = 8) were injected with same value of DMSO. MRI (Broker Biospec 7.0 T Imaging System) was performed to detect tumor dimensions, and the body weights of mice were also check ed.Tumor value = (π/6) × length × width × depth, All animal experiments were performed in accordance with the Guidelines for the Care and Use of Laboratory Animals, and all experimental protocols were approved by the Institutional Animal Care and Use Committee of Army Medical University.

### Statistical analysis

All experiments were performed in triplicate. Statistical analyses were carried out by using SPSS 13.0 statistical software. Data were exhibited as means ± SD. Statistical significance was calculated by Student’s *t* test or one-way ANOVA. Survival was analyzed by Kaplan–Meier method and compared by log-rank test by GraphPad Prism 5. (*) *p* < 0.05 was considered statistically significant.

## Results

### TRPV4 expression is elevated in malignant glioma and has a negative correlation with the prognosis of glioma patients

**Figure 1:**
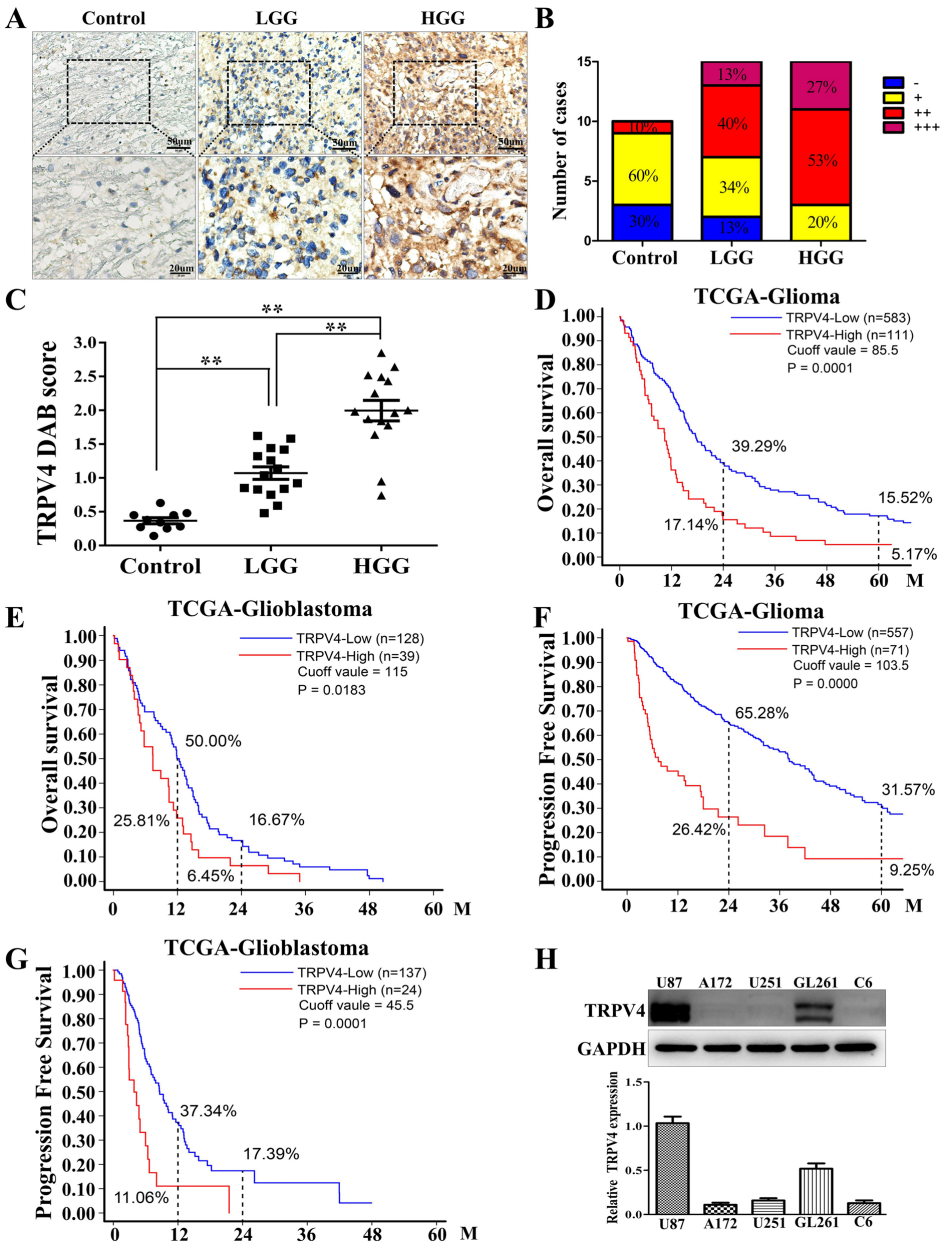
TRPV4 is highly expressed in malignant glioma, and higher expression indicates poor prognosis in glioma patients. (**A**) The expression of TRPV4 in normal brain tissues (control), low-grade glioma (LGG) and high-grade glioma (HGG) tissues was detected by IHC, scale bar = 50 µm or 20 µm. (**B**) The percentage of TRPV4 staining grades (− , + , +  + , +  + +) in controls, LGG and HGG. (**C**) IHC staining for TRPV4 was assessed semiquantitatively. TRPV4 intensity and the percentage of positive cells were multiplied to give a weighted score for each case. Data are plotted as the means ± SD from control (0.37 ± 0.14, n = 10), LGG (1.07 ± 0.36, n = 15) and HGG (1.99 ± 0.59, n = 15), **p < 0.01. (**D**) Kaplan–Meier analysis of the 2-year and 5-year overall survival (OS) rates of TRPV4low patients and TRPV4high patients from the TCGA-glioma dataset. (**E**) Kaplan–Meier analysis of the 1-year and 2-year OS rates of TRPV4low and TRPV4high patients from the TCGA-glioblastoma dataset. (**F**) Kaplan–Meier analysis of 2-year and 5-year progression free survival (PFS) rates of TRPV4low patients and TRPV4high patients from the TCGA-glioma dataset (**G**) Kaplan–Meier analysis of 1-year and 2-year PFS rates of TRPV4low patients and TRPV4high patients from the TCGA-GBM dataset. All *p* values were determined using the log-rank test. (**H**) The expression of TRPV4 in glioblastoma cell lines (C6, U87, A172, U251, GL261) was detected by western blotting.

To investigate whether TRPV4 is a biomarker for glioma, we first detected the expression of TRPV4 in normal brain tissues and different grades of glioma by IHC assays. The results showed weak TRPV4 staining in normal brain tissues (control), and TRPV4 was predominantly observed in the membrane and cytoplasm of tumor cells of low-grade glioma (LGG, WHOI and II). In contrast, strong TRPV4 staining was observed in the cytoplasm and membrane of high-grade glioma (HGG, WHO III and IV) (Fig. 1A). The distribution of TRPV4 intensity among the control, LGG and HGG groups is shown in Fig. 1B. Eighty percent of HGG showed moderate to intense (+ + to +  + +) TRPV4 staining, in contrast to LGG (53%) and control (10%). The intensity of TRPV4 staining appeared to be correlated with malignant progression. Semiquantitative analysis of TRPV4 immunostaining showed that the difference in TRPV4 score reached statistical significance in HGG compared to LGG and control (Fig. 1C). Next, to test the possibility of TRPV4 as a prognostic marker for glioma patients, we analyzed the TRPV4 mRNA levels in the TCGA-glioma dataset. Kaplan–Meier analysis of the overall survival (OS) rates showed that the 2-year and 5-year OS rates for the low-expression group (TRPV4^low^) were significantly higher than those for the high-expression group (TRPV4^high^) (*p* = 0.0001) (Fig. 1D). The 1-year and 2-year OS rates for TRPV4^low^ patients were significantly higher than those for TRPV4^high^ patients with glioblastoma (*p* = 0.0183) (Fig. 1E). Furthermore, we investigated the correlation between TRPV4 and the progression-free survival (PFS) rate of patients in the TCGA-glioma dataset. The results showed that the 2-year and 5-year PFS rates in TRPV4^low^ patients were significantly higher than in TRPV4^high^ patients. (*p* = 0.0000) (Fig. 1F). Similarly, in the glioblastoma dataset. The 1-year and 2-year PFS rates in TRPV4^low^ patients were significantly higher than in TRPV4^high^ patients (*p* = 0.0001) (Fig. 1G). However, the expression level of TRPV4 had no influence on the OS or PFS of LGG patients (data not shown). Therefore, we concluded that TRPV4 expression was elevated in HGG and had a negative correlation with the prognosis of patients with glioma, especially GBM. In vitro, we detected the expression of TRPV4 in five glioblastoma cell lines (U87, A172, U251, GL261 and C6) and found that TRPV4 was only expressed in U87 and GL261 cells (Fig. 1H). Notably, two bands of TRPV4 protein were detected in glioblastoma cells by western blot, which implied that TRPV4 protein underwent some modification in glioblastoma cells, such as phosphorylation or ubiquitylation.

### TRPV4 promotes glioblastoma cell migration and invasion in vitro

**Figure 2:**
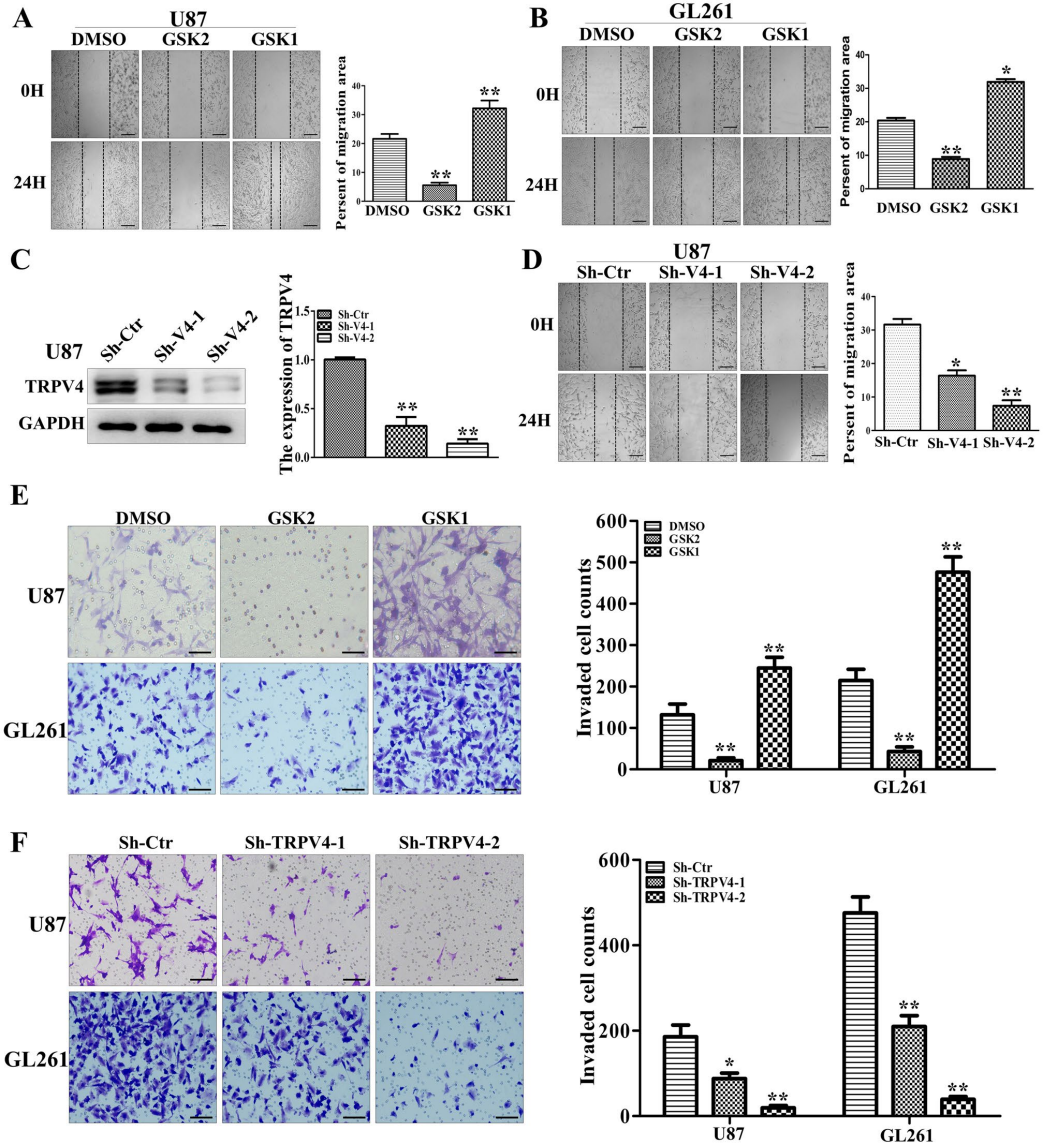
TRPV4 promotes the migration and invasion ability of glioblastoma cells in vitro. (**A–B**) U87 and GL261 cells were treated with the TRPV4 antagonist GSK2 (50 nM) or the agonist GSK1 (10 nM). Cells were scratched and imaged immediately (0 h) and after 24 h (left). Relative motility was quantified by measuring the cell surface area using ImageJ, scale bar = 100 µm. (**C**) The downregulation effect of two shRNAs targeting TRPV4 (sh-TRPV4-1 and sh-TRPV4-2) was detected by western blot assays. (**D**) The migration ability of U87 cells after TRPV4 downregulation was detected by wound-healing assay, scale bar = 100 µm. (**E**) Representative result (left) and histogram (right) of Transwell assays when cells were treated with DMSO, GSK2 (50 nM) or GSK1 (10 nM) in U87 and GL261 cells, scale bar = 50 µm. (**F**) Representative results (left) and histogram (right) of Transwell assays when TRPV4 was silenced by shRNA in U87 and GL261 cells, scale bar = 50 µm. Student’s *t* test was used for statistical analysis. *p < 0.05, **p < 0.01.

TRPV4 was upregulated in HGG and indicated poor outcomes in glioblastoma patients, which implies that TRPV4 may promote glioblastoma progression. To test this hypothesis, we first investigated the migration of glioblastoma cells after treatment with the TRPV4 agonist GSK1016790A (GSK1, 10 nM) or the antagonist GSK2193874 (GSK2, 100 nM). The results from wound-healing assays showed that the gap sizes after 24 h in the GSK2 group were markedly wider compared to the control group, whereas the gap sizes in the GSK1 group were significantly closer than the control group in U87 cells (Fig. 2A). Similar results were obtained from the GL261 cell line (Fig. 2B). Next, we used two different shRNAs to interfere with TRPV4 expression in U87 cells and obtained approximately 60% and 90% knockdown effects at the protein level (Fig. 2C). Downregulation of TRPV4 also distinctly impaired the migration ability of U87 cells (Fig. 2D). We repeated above assays and obtained similar results in GL261 cells (Fig. S1A,B). Next, we performed Transwell assays in U87 and GL261 cells after GSK2 or GSK1 treatment and observed that the number of cells that invaded toward the pores and Matrigel was significantly decreased after TRPV4 repression but significantly increased after TRPV4 activation compared to DMSO (Fig. 2E). Likewise, knockdown of TRPV4 reduced the number of invading cells across the Transwell membrane filter compared to the control in two glioblastoma cell lines (Fig. 2F). Transwell assays with U251 cells were employed as a negative control because they barely expressed TRPV4 protein, and we found that neither the same dose of TRPV4 agonist or antagonist nor sh-TRPV4 treatment affected the invasion ability of U251 cells (Fig. S1C,D). Simultaneously, we tested whether TRPV4 could mediate the proliferation and apoptosis of glioblastoma cells. The results from flow cytometry assays and CCK-8 assays showed that TRPV4 agonist (GSK1, 10 nM) and antagonist (GSK2, 100 nM) did not impact the cell cycle or apoptosis of GBM cells (Fig. S1E-G). Thus, we concluded that the promotion of cell invasiveness might be the major reason of TRPV4 upregulation leads to poor prognosis in glioblastoma patients.

### TRPV4 interacts with F-actin at cellular protrusions of glioblastoma cells

**Figure 3:**
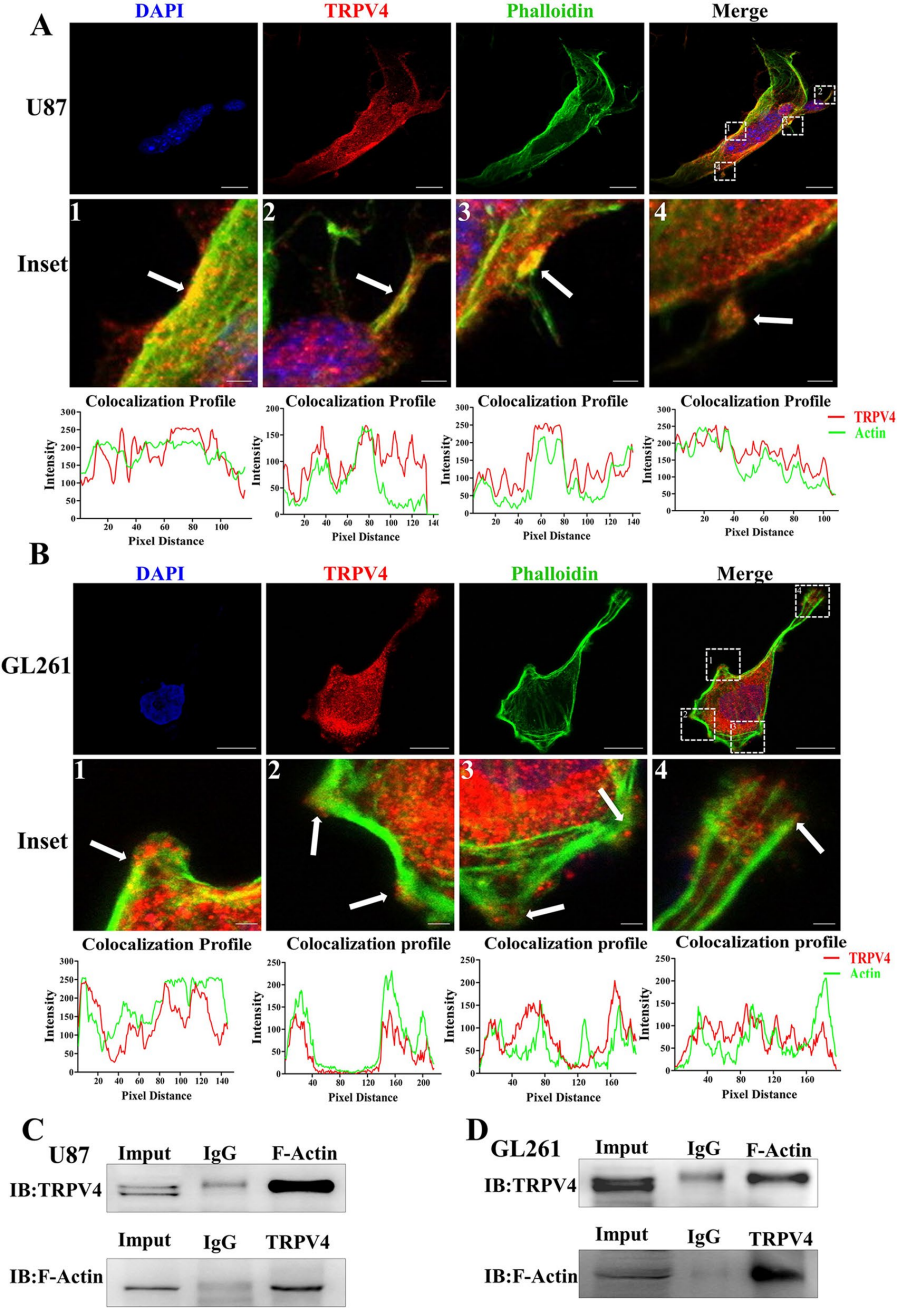
TRPV4 colocalizes and interacts with cytoskeletal protein F-actin. (**A**) U87 cells were plated on dishes for IF, and F-actin was labeled with FITC-phalloidin (green). TRPV4 was labeled with anti-TRPV4 antibody followed by TRITIC-conjugated antibody (red). Inset images show the TRPV4 distribution at the membrane and cellular protrusions. The colocalization profiles of inset images were analyzed by ImageJ and are listed below the image. Scale bar = 20 µm and 2 µm. (**B**) GL261 cells on confocal dishes for F-actin and TRPV4 staining to show colocalization. The inset images and colocalization profiles are listed below. Scale bar = 10 µm and 1 µm. (**C**-**D**) Coimmunoprecipitation of TRPV4 and F-actin: the whole-cell lysate was immunoprecipitated (IP) with anti-TRPV4 or F-actin antibody, and the immunocomplexes were immunoblotted (IB) with the opposite antibody in U87 and GL261 cells.

TRPV4 promotes glioblastoma progression by accelerating cell invasion and eventually leads to poor prognosis. To test the underlying mechanism of TRPV4 in these processes, we first utilized IF to show the localization of TRPV4 in glioblastoma cells. We found that TRPV4 was localized at the plasma membrane and cytoplasm in U87 cells. F-actin, shown by FITC-phalloidin staining, was predominantly located in the cell membrane, membrane ruffles and cell protrusions. In the merged images, we surprisingly observed that these structures showed a strong colocalization of TRPV4 with F-actin (white arrow in the inset image 1–4), whereas the intracellular part of TRPV4 clearly lacked colocalization with stable actin structures, such as actin bundles and stress fibers (Fig. 3A). We also found TRPV4 co-expression with F-actin at the cell membrane, membrane ruffles and cellular protrusions in GL261 cells (white arrows in inset image 1–4), (Fig. 3B). These data indicated that TRPV4 co-localized with F-actin at the cellular protrusions and membrane, which may be responsible for the cell migration and invasion of glioblastoma cells. Next, we employed co-IP experiments with TRPV4 antibodies in U87 cells and observed that TRPV4 antibodies precipitated TRPV4 together with F-actin proteins. Conversely, F-actin antibodies also precipitated F-actin together with TRPV4 protein (Fig. 3C). To strengthen our observations, we repeated co-IP in GL261 cells and observed similar results (Fig. 3D). Above results indicated that a strong colocalization and interaction between TRPV4 and F-actin existed in glioblastoma cells.

### TRPV4 promotes cellular protrusions formation in glioblastoma cells

**Figure 4:**
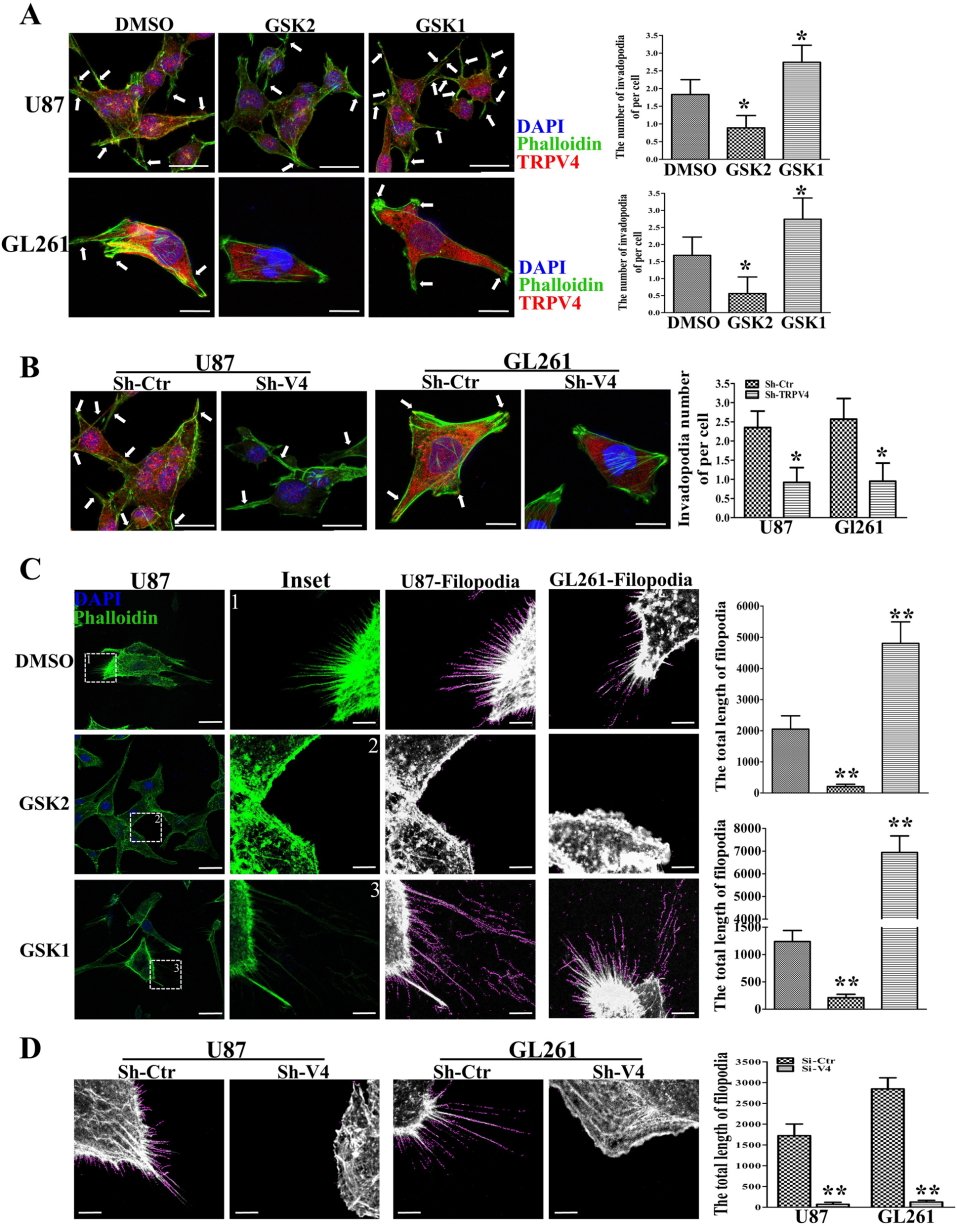
TRPV4 strengthens invadopodia and filopodia formation in glioblastoma cells. (**A**) Representative results of cell morphology and invadopodia were altered by TRPV4 antagonist GSK2193874 (GSK2, 100 nM) or TRPV4 agonist GSK1016790A (GSK1, 10 nM) treatment in U87 and GL261 cells. Histogram showing the number of invadopodia per cell, scale bar = 20 µm and 10 µm, respectively. (**B**)The cell morphology and invadopodia were changed after down-regulation of TRPV4 in U87 and GL261 cells, scale bar = 20 µm and 10 µm, respectively. (**C**) IF shows filopodia changes after U87 and GL261 cells were treated with TRPV4 antagonist or agonist, Scale bar = 20 µm. The inset images were analyzed by Fiji software, Filopodia are shown with violet line, Scale bar = 4 µm. The total length of filopodia were calculated and shown by the histogram. (**D**)The filopodia was altered by TRPV4 down-regulation in U87 and GL261 cells. Histograms showing the total length of filopodia. The Fluorescent images were exported by Zen software and analyzed filopodia by Fiji software. Scale bar = 4 µm. *p < 0.05, **p < 0.01.

The strong co-expression of TRPV4 and F-actin made us wonder whether TRPV4 regulates the formation cellular protrusions, such as invadopodia and filopodia. We utilized IF to examine the influence of TRPV4 on cellular protrusions. As shown in Fig. 4A, TRPV4 localized to the cytoplasm, F-actin rich membrane and protrusions in the control group, and cells displayed a well-organized cytoskeleton and maintained the middling length of invadopodia at the dual terminals. TRPV4 antagonist treatment sharply reduced the number of invadopodia. However, TRPV4 agonist significantly increased the number of invadopodia in U87 and GL261 cells. To strengthen our observations, we also investigated the change in cellular invadopodia when TRPV4 was downregulated by shRNA, as shown in Fig. 4B. TRPV4 was sharply downregulated compared to the control group, and the number of invadopodia was significantly reduced both in U87 and GL261 cells. In an attempt to test whether TRPV4 also regulates filopodia formation, we first blocked TRPV4 by GSK2 in U87 cells. The total length of filopodia was significantly reduced. In contrast, the total length filopodia were significantly increased after GSK1 treatment. The similar trend was observed in GL261 cell. (Fig. 4C). sh-TRPV4 transfection also repressed filopodia formation in U87 and GL261 cells (Fig. 4D). The above results demonstrated that TRPV4 was crucial for invadopodia and filopodia formation in glioblastoma cells.

### TRPV4 regulates Cdc42/N-wasp axis activation in glioblastoma cells

**Figure 5:**
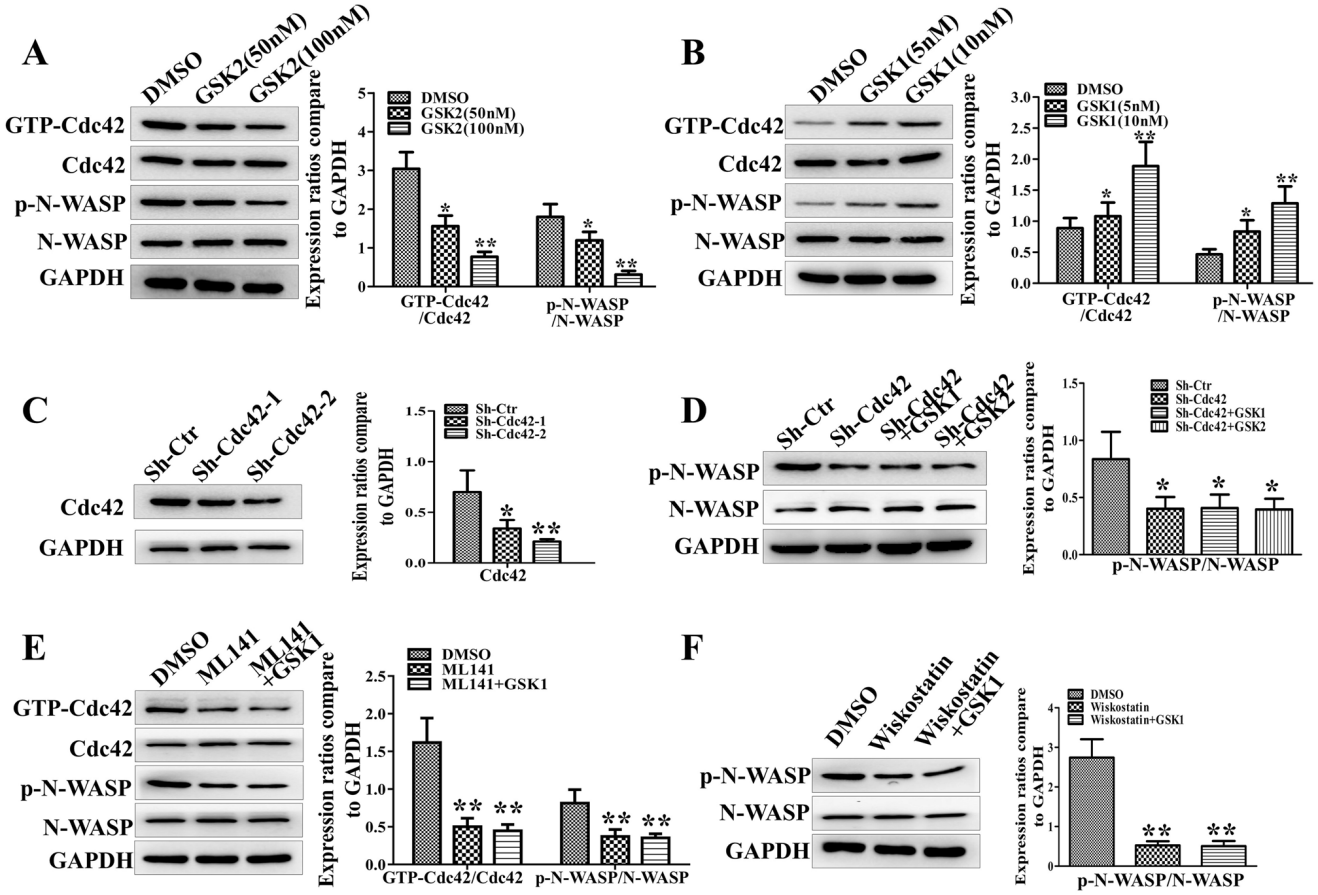
TRPV4 regulates Cdc42/N-wasp axis in glioblastoma cells. (**A**–**B**) Pull-down and western blot assays showing Cdc42/N-wasp axis activation in U87 cells treated with antagonist (50 nM or 100 nM) or agonist (5 nM or 10 nM). (**C**) Western blot assays show the knockdown effect of two sequences of sh-Cdc42 in U87 cells. (**D**) The activation of N-wasp was detected by western blot after knockdown Cdc42 or combined with GSK2 and GSK1. (**E**) Western blot analysis of the activation of the Cdc42/N-wasp axis after ML141 (Cdc42 inhibitor, 500 nM) treatment or combined with TRPV4 agonist. (**F**) The activation of N-wasp was detected by western blot assay after treatment with wiskostatin (N-wasp inhibitor, 5 µM) without or with TRPV4 agonist. *p < 0.05, **p < 0.01.

Previous literature has demonstrated that the Cdc42/N-wasp axis influences F-actin assembly and cell invasion in several types of cancer. We first employed pull-down and western blot assays to detect the influence of TRPV4 on the activation of Cdc42/N-wasp signaling. U87 cells were treated with 50 nM or 100 nM GSK2 or left untreated. The total levels of Cdc42 and N-wasp were not significantly altered by GSK2 treatment. However, GSK2 inhibited the activation of Cdc42 and N-wasp in a dose-dependent manner (Fig. 5A). In contrast, Cdc42 and N-wasp were activated in a dose-dependent manner when TRPV4 was stimulated with different doses of GSK1 (Fig. 5B). We also knocked down Cdc42 by shRNA. Indeed, targeted shRNA treatment clearly decreased the expression of Cdc42 and the activation of N-wasp. The repression of N-wasp, caused by Cdc42 knockdown, was not significantly altered by TRPV4 antagonist or agonist treatment (Fig. 5C&5D). Furthermore, the Cdc42 inhibitor ML141 significantly repressed the activation of the Cdc42/N-wasp axis in U87 cells. GSK1 treatment couldn’t alleviate the repression of the N-wasp caused by Cdc42 inhibition (Fig. 5E). Next, we found that an N-wasp inhibitor (wiskostatin) repressed the activation of N-wasp, and GSK1 also couldn’t rescue the repression of N-wasp in U87 cells caused by wiskostatin (Fig. 5F). We concluded here that TRPV4 regulates Cdc42/N-wasp axis in glioblastoma cells.

### Cdc42/N-wasp axis is crucial for TRPV4-mediated invasion and cellular protrusions of glioblastoma cells

**Figure 6:**
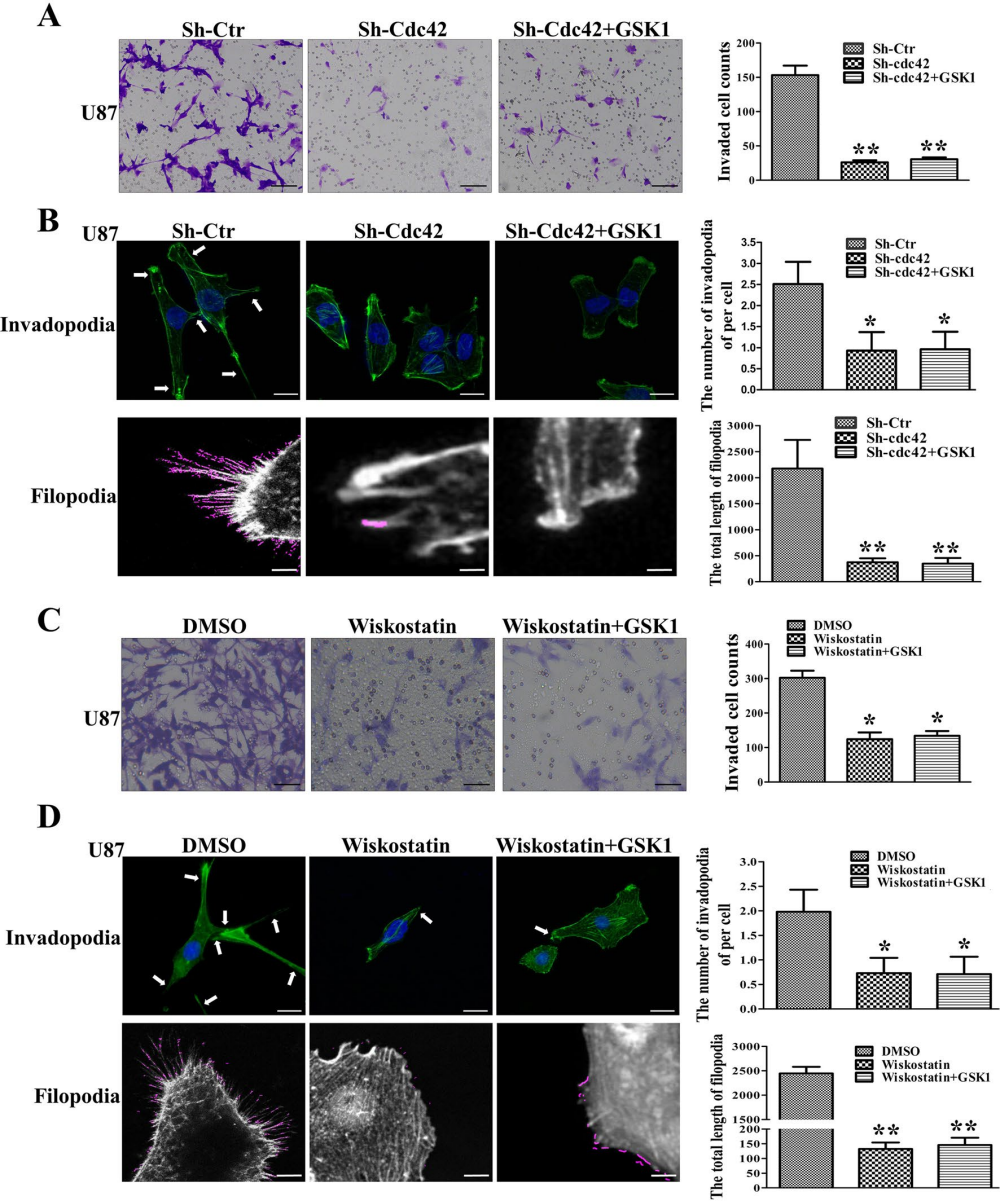
Cdc42/N-wasp axis is crucial for TRPV4-mediated protrusion formation and invasion in glioblastoma cells. (**A**) Transwell assays show the invasion ability after Cdc42 downregulation or combined with TRPV4 agonist treatment in U87 cells, scale bar = 100 µm. (**B**) U87 cell invasion ability was detected by Transwell assays after N-wasp inhibitor (wiskostatin, 5 µM) treatment without or with TRPV4 agonist. Scale bar = 100 µm. (**C**) Cellular invadopodia and filopodia changed after Cdc42 down-regulation or combined with TRPV4 agonist treatment in U87 cells. Histograms show the number of invadopodia per cell and the total length of filopodia, Scale bar = 10 µm and 4 µm. (**D**) Cellular protrusions changed after N-wasp repression or combined with GSK1 treatment in U87 cells. Histograms show the number of invadopodia per cell and the total length of filopodia, Scale bar = 10 µm and 4 µm. The Fluorescent images were exported by Zen software and analyzed filopodia by Fiji software. *p < 0.05, **p < 0.01.

To test whether the Cdc42 mediates the function of TRPV4 in regulating the invasiveness of glioblastoma, we performed Cdc42 inhibitor to treated U87 cell. ML141 addition sharply decreased the invaded cell compared to control. And TRPV4 agonist could not reverse the repression of invasion caused by ML141 (Fig. 6A). Likewise, we asked whether Cdc42 transduces TRPV4 effect to cellular protrusions. We found that downregulation of Cdc42 inhibited cell invadopodia and filopodia formation in U87 cells, and TRPV4 stimulation failed to rescue the depression of invadopodia and filopodia formation when Cdc42 was knocked down (Fig. 6B). Thus, Cdc42 is vital for TRPV4-mediated invasion and protrusions formation of glioblastoma. Next, we investigated the function of N-wasp in TRPV4-mediated invasion in glioblastoma. N-wasp inhibitor reduced the number of invaded cells of U87, and the promotion function of GSK1 was blocked by Wiskostatin addition(Fig. 6C). Furthermore, repression of N-wasp decreased the formation of invadopodia and filopodia, and GSK1 treatment did not alleviate the repression by N-wasp depression (Fig. 6D). These results suggested that the Cdc42/N-wasp axis mediated the TRPV4 function in cell invasion by altering cellular protrusions.

### Blocking TRPV4 inhibits glioblastoma invasion-growth in vivo

**Figure 7:**
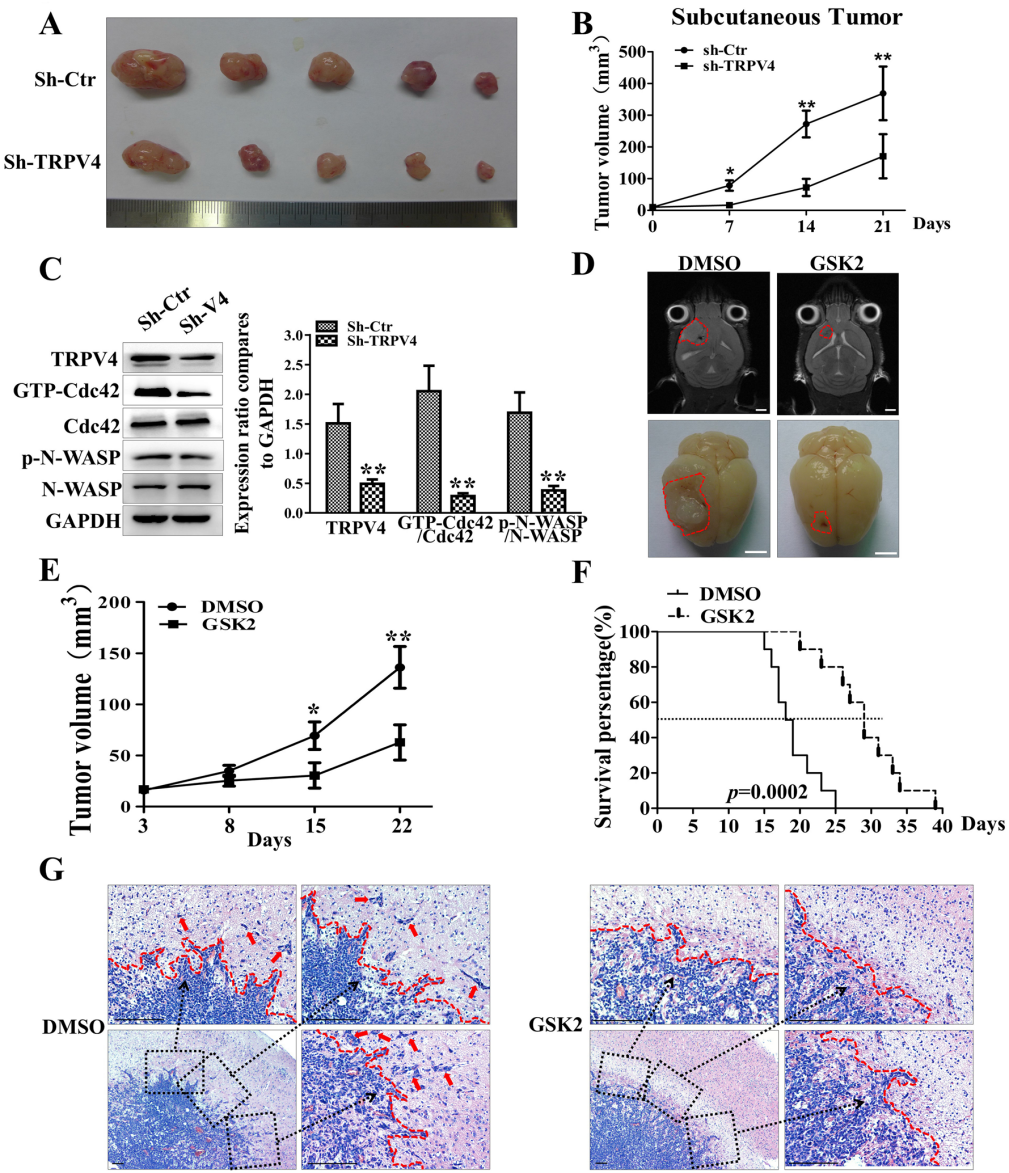
Blocking TRPV4 impairs the invasion-growth of glioblastoma in vivo. (**A**) The gross observation of a subcutaneous tumor at 21 days postinjection after dissection from the groin (upper: sh-Ctr; lower: sh-TRPV4). (**B**) The volume of subcutaneous tumor was calculated weekly after implantation. (**C**) Western blot showing the expression of TRPV4 and the activation of the Cdc42/N-wasp axis in subcutaneous glioblastoma tissues. (**D**) Representative MRI images (scale bar = 5 mm) and gross observations (scale bar = 5 mm) of orthotopic xenograft glioblastoma from the DMSO and GSK2 groups. (**E**) The tumor value of intracranially transplanted glioblastoma with micro-MRI detection at 3, 8, 15, and 22 days after treatment. (**F**) The survival duration of the mice was monitored in the indicated groups, and the survival time was analyzed by the Kaplan–Meier method and compared by the log-rank test. (**G**) H&E staining of orthotopic glioblastoma xenografts to explore the invasion states of glioblastoma in the DMSO and GSK2 groups., scale bar = 100 μm. *p < 0.05, **p < 0.01.

**Figure 8:**
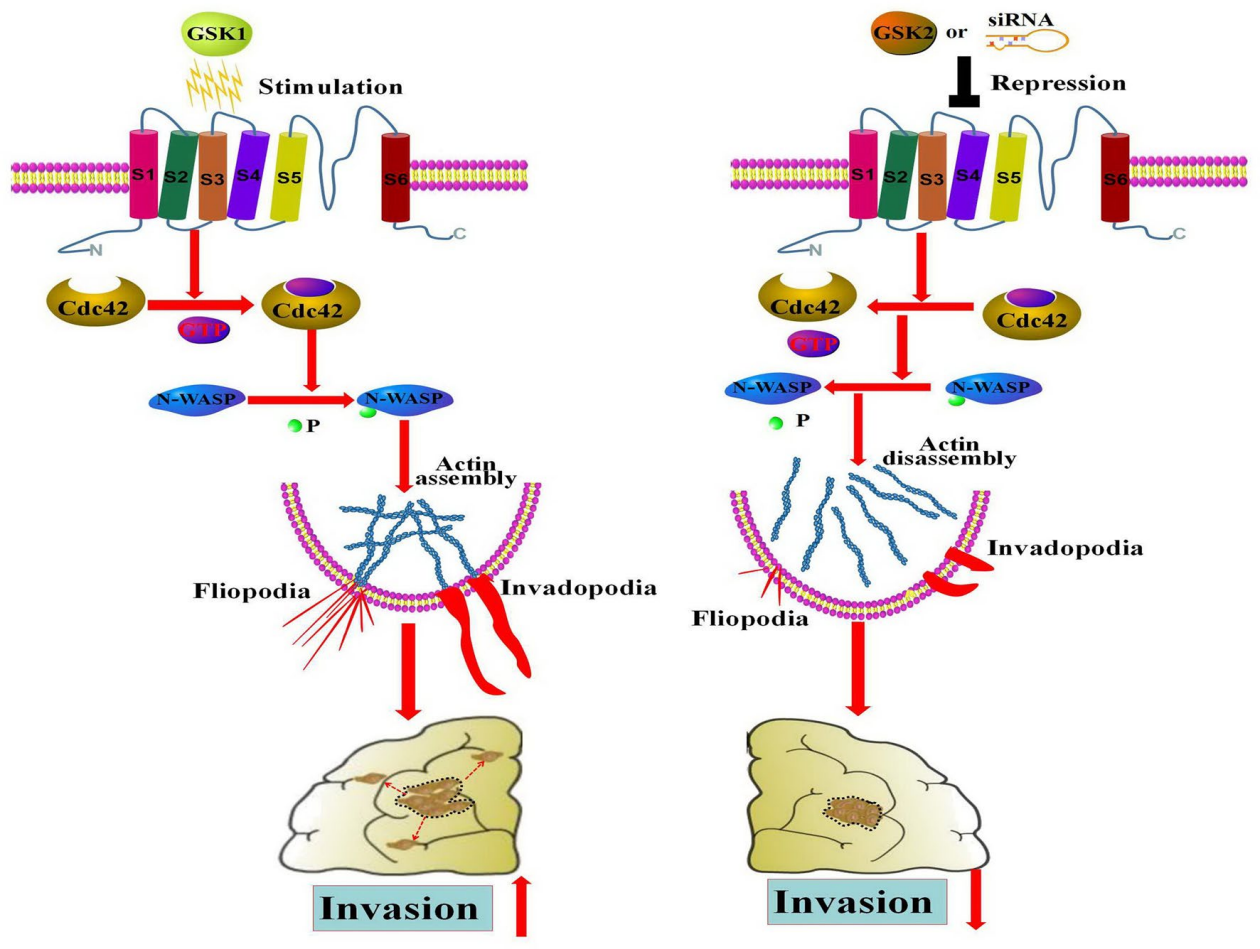
Schematic model: TRPV4 promotes protrusions (invadopodia and filopodia) formation and invasion through the Cdc42/N-wasp axis in glioblastoma. The schematic model was drawn with pathway builder 2.0 software.

To verify the functions of TRPV4 in the pro-invasion of glioblastoma in vivo, stably TRPV4-silenced and control cells were subcutaneously implanted into the left (sh-Ctr) and right (sh-TRPV4) groins of each nude mouse. We found that repression of TRPV4 significantly slowed the subcutaneous tumor invasion-growth compared to the control group (Fig. 7A,B). The expression of TRPV4 and the activation of the Cdc42/N-wasp axis were clearly decreased in the sh-TRPV4 groups (Fig. 7C). Next, we established an intracranial glioblastoma model by injecting U87 cells at the right striatum of nude mice and detected tumor volume by MRI, and we found that the TRPV4 inhibitor significantly reduced the volumes of tumors compared to the DMSO group (Fig. 7D,E). GSK2 treatment significantly prolonged the survival time of glioblastoma-bearing mice compared to DMSO (Fig. 7F). HE staining of intracranial tumors showed that GSK2 impaired the invasion capacity of glioblastoma in the encephalic nude mouse model. We calculated the length of the tumor border line, the number of tumor cells far from the tumor border, and the total and mean distance of tumor cells from the border line. The above indices were all significantly decreased with GSK2 treatment (Fig. 7G, S2A). However, the TRPV4 antagonist treatment did not change the percentage of Ki-67-positive cells in intracranial glioblastoma tissues (Fig. S2B), which implied that GSK2 treatment did not affect the proliferation of glioblastoma in vivo. These results indicated that TRPV4 activated the cdc42/N-wasp axis to regulate the protrusion formation and invasion of glioblastoma, whereas blocking TRPV4 with an inhibitor or shRNA effectively repressed glioblastoma invasion in vitro and in vivo (Fig. 8).

## Discussion

In this study, we have shown that the actin-bundling protein TRPV4 is expressed in normal brain tissues, glioma tissues and permanent glioblastoma cell lines. Interestingly, TRPV4 expression increased with the malignancy grade of glioma. TRPV4 expression level was negatively correlated with prognosis in patients with all grades of glioma and glioblastoma multiforme thus, we concluded that TRPV4 could be a biomarker and prognostic factor for glioblastoma patients.

Increasing studies associate TRPV4 with multiple processes of cancer. First, recent work has started to highlight the role of TRPV4 in angiogenesis. Arachidonic acid (AA)-based calcium entry has been linked to angiogenesis in breast-derived tumor endothelial cells (TECs), and TRPV4 is upregulated in breast TECs compared to healthy endothelial cells. TRPV4 was stimulated by AA, and the subsequent calcium conductance was correlated with increased migration of breast TECs by actin remolding^17^. In contrast, TECs from a transgenic adenocarcinoma mouse prostate model exhibited low levels of TRPV4 expression compared to normal endothelial cells. Downregulation of TRPV4 was related to extracellular matrix stiffness and increased migration of endothelial cells and abnormal angiogenesis. The absence of TRPV4 induced increased vascular density, vessel diameter and reduced pericyte coverage^20^. In another study, Thoppil, R J et al. demonstrated that the absence of TRPV4 in TECs led to a significant increase in proliferation, migration, and abnormal tube formation in vitro. There was enhanced angiogenesis and vascular growth in vivo in TRPV4 KO mice^21^. The conflicting results may be explained by the different methods used to study TRPV4. The data suggest that basal levels of TRPV4 activation may inhibit angiogenesis, whereas overactivation of TRPV4 by an agonist or mechanical stimulation may increase angiogenesis.

Second, previous research has shown that TRPV4 mediates calcium-sensing receptor function in regulating the migration and invasion of gastric cancer cells via Ca(2 +)/AKT/beta-catenin^22^. TRPV4 may regulate breast cancer metastasis by regulating cell softness through the Ca(2 +)-dependent AKT-E-cadherin signaling axis^16^. AKT was upregulated by TRPV4 in both studies, and AKT activation was strongly involved in gastric and breast tumor metastasis, as in prior studies. TRPV4 regulates a network of proteins involved in the cytoskeleton (β-tubulin, epsilon and talin) and extracellular matrix remodeling (fibronectin, clusterin and osteopontin)^16^. Another study showed TRPV4 regulates breast cancer cell migration by increasing cell deformability^23^. TRPV4 changes expression in dorsal root ganglia after cancer cell inoculation and may play a role in transducing cancer-induced hyperalgesia^24^. Our study demonstrated that TRPV4 mediated glioma cell migration and invasion, which strengthens the idea that TRPV4 is essential for tumor invasiveness.

We reported that TRPV4 interacted with actin and regulated actin arrangement to alter the formation of invadopodia and filopodia in glioblastoma in this study, which is consistent with previous research. Becker et al. reported that TRPV4 is found in CHO cells at highly dynamic structures, including microvilli, filopodia, and lamellipodia edges. TRPV4 interacts with F-actin and functions in sensing hypotonicity and in the development of regulatory volume decrease (RVD)^25^. Another study suggested that TRPV4 localized to apical microvilli in a small subset of human fetal retinal pigment epithelia^26^. Further, TRPV4 channels appear polarized and enriched in lamellipodial structures in GnRH-secreting neurons, and activation of TRPV4 leads to retraction of the lamellipodia and decreased migratory behavior of cells^27^. The C-terminus of TRPV4 interacts directly with tubulin and actin, which compete for binding. The interaction with TRPV4 stabilizes microtubules. Activation of TRPV4 induces remarkable morphological changes affecting lamellipodial, filopodial, growth cone, and neurite structures in nonneuronal cells, DRG-neuron-derived F11 cells, and IB4-positive DRG neurons^28^. Alpha/beta tubulin heterodimerizes to make microtubules, a vital process in the cytoskeleton that plays important roles in cellular structural support, intracellular trafficking and cell division^29^. Tubulin beta-5 is one subtype of tubulin found in vertebrates that assembles to form cellular microtubules. TRPV4 was found to colocalize and form a complex with tubulin beta 5 in transfected HEK293 cells^30^. An association of tubulin beta-5 with TRPV4 has also been found in DRG neurons and plays a role in the mechanosensation in neuropathic pain conditions^31^.

Our results suggest that the Cdc42/N-wasp axis conducts the function of TRPV4 in protrusion formation and invasion. Cdc42 is a small GTPase that switches between an inactive GDP-bound state and an active GTP-bound state. Cdc42 is regulated by guanine nucleotide exchange factors (GEFs), GTPase activating proteins (GAPs) and guanine nucleotide dissociation inhibitors (GDIs)^32^. Cdc42 has been found in multiple human cancers and is implicated in tumor growth, cell-cycle progression, epithelial-mesenchymal transition, angiogenesis, oncogenic transformation, and tumor migration/invasion^12^. Cdc42 exerts its function by activating downstream adaptors/effectors, including N-wasp. In glioma, the expression of N-WASP is upregulated and plays key roles in low-oxygen-induced accelerated brain invasion^33^. Meanwhile, Mair et al. and Liu et al. reports that activation of Arp2/3 by N-wasp is essential for actin polymerization during migration of glioma cells^34,35^. A more recent study indicated that N-wasp mediates the function of RTVP-1 in regulating glioma cell migration and invasion via matrix degradation and invadopodia formation^36^. We report that Cdc42 regulates the phosphorylation of N-wasp to manage the formation of filopodia and invadopodia. We first related TRPV4 to the Cdc42/N-wasp pathway in glioblastoma and explored the area of the TRP family with cellular protrusion formation. This Ca^2+^-permeable channel regulated by endogenous Ca^2+^ has been studied in endothelial cells, keratinocytes, and DRG neurons^14^. Furthermore, TRPV4 directly regulates Ca^2+^ influx and increased cell migration of metastatic breast cancer cells^16^. Activation of Cdc42 is critical for antigen-stimulated pathways leading to Ca^2+^ mobilization in RBL mast cells^37^. Thus, we hypothesize that TRPV4 targets the Cdc42/N-wasp signal via Ca^2+^ influx, which will be investigated in our next study.

## Conclusion

In summary, TRPV4 blockade in glioblastoma cells results in decreased invadopodia and filopodia formation and decreased invasion, suggesting that TRPV4 plays a key role in glioblastoma invasiveness. The molecular mechanisms by which TRPV4 interacts with actin via Cdc42/N-wasp to alter glioblastoma protrusions, the use of a stable TRPV4 knockdown system and the development of pharmacological inhibitors (GSK2193874) of TRPV4 will lead to a premising therapeutic agent for anti-invasion in human glioblastoma.

## Supplementary information

Supplementary Information.

## Abbreviations

Cdc42: Cell division cycle 42
N-wasp: Neural Wiskott–Aldrich syndrome proteins
mDIA: Mammalian homolog of diaphanous
WAVE: Wiskott–Aldrich syndrome protein family verprolin homologous protein
CHO cell: Chinese hamster ovary cell
DRG cell: Dorsal root ganglia cell
RTVP-1: Related to testis-specific, vespid and pathogenesis protein 1
