# Supplementary material for: TRPV4 activates the Cdc42/N-wasp pathway to promote glioblastoma invasion by altering cellular protrusions
Source: Sci Rep. 2020 Aug 25;10:14151. doi: 10.1038/s41598-020-70822-4 (PMC7447819; doi:10.1038/s41598-020-70822-4)
Supplement: Supplementary file 1 — Supplementary Information. [file 41598_2020_70822_MOESM1_ESM.pdf]

TRPV4 activates the Cdc42/N-wasp pathway to promote glioblastoma invasion by altering cellular protrusions

Wei Yang [1#], Peng-fei Wu [2#], Jian-xing Ma[2], Mao-jun Liao [2], Lun-shan Xu[2,*], and Liang Yi [2,*]

Figure 1

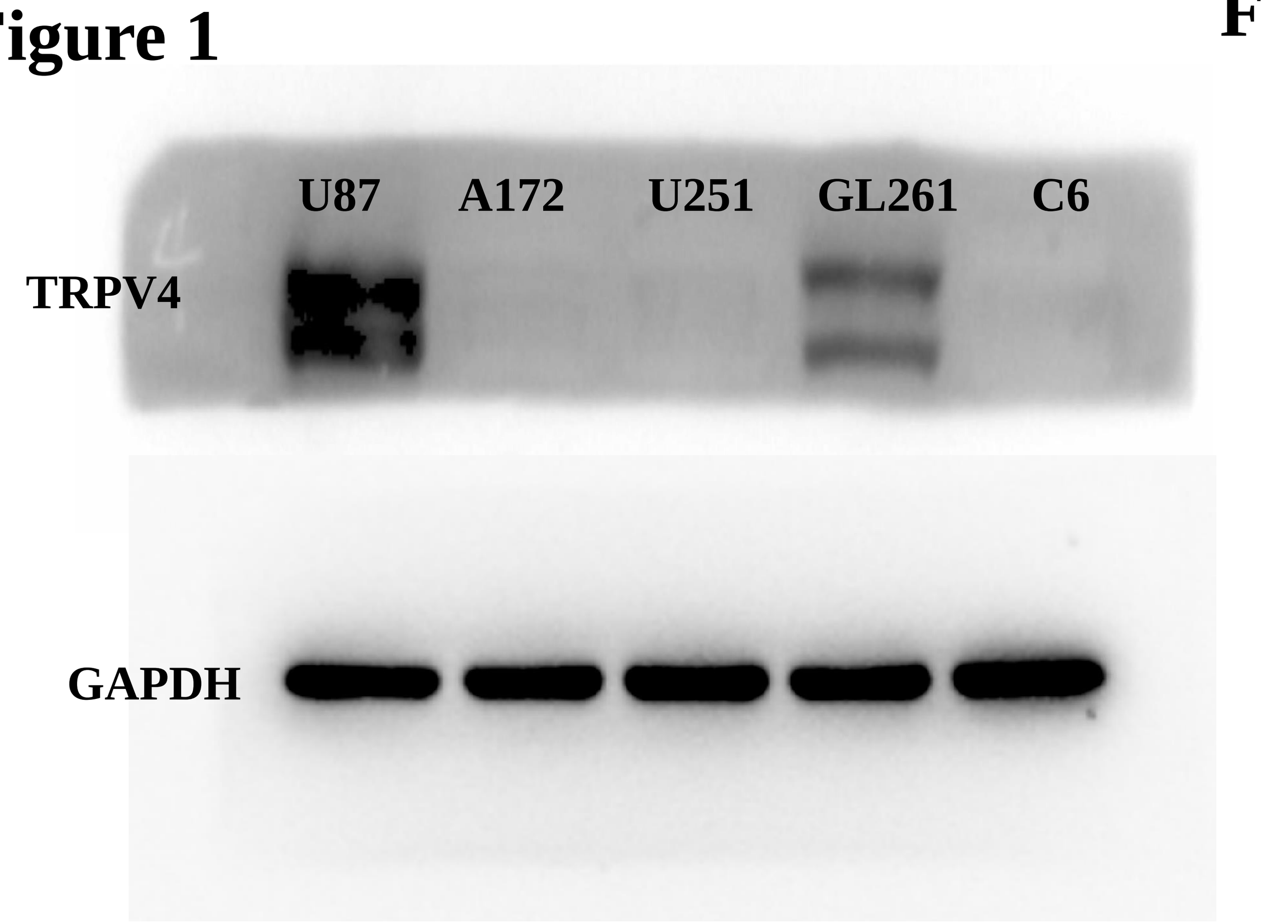

Figure 2

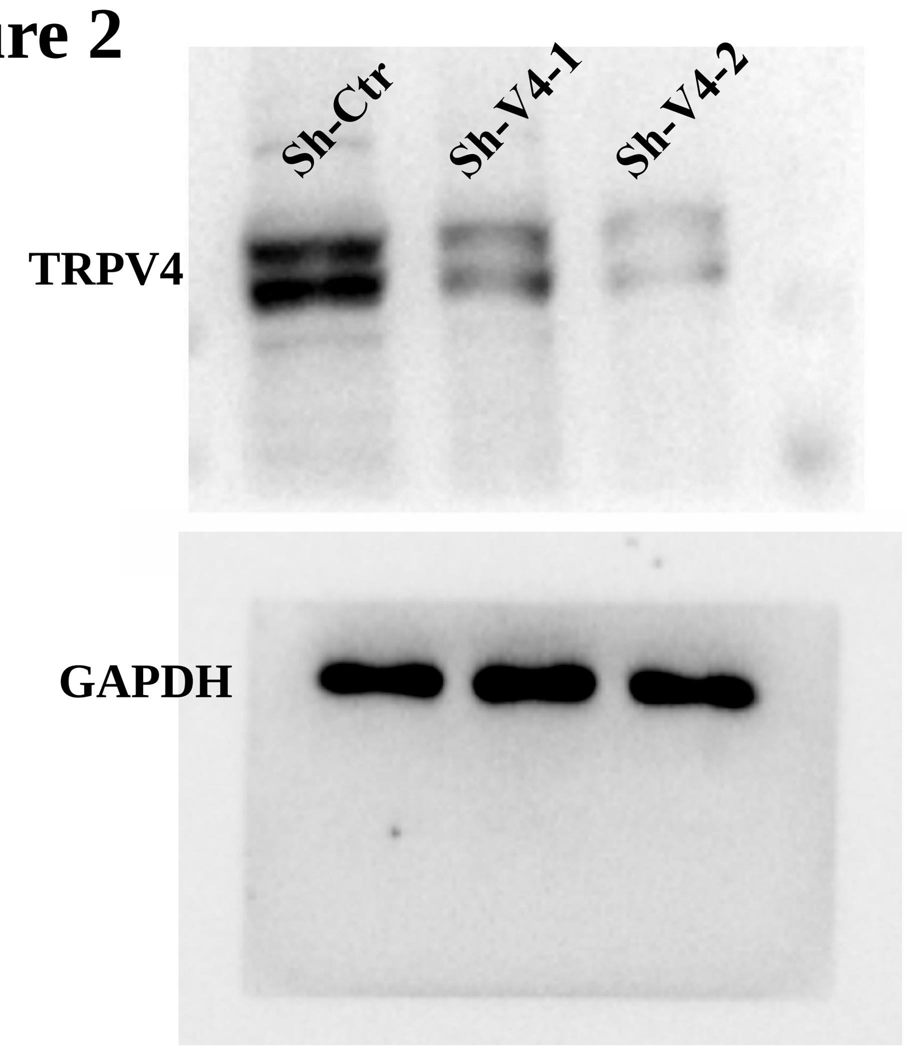

Figure 3

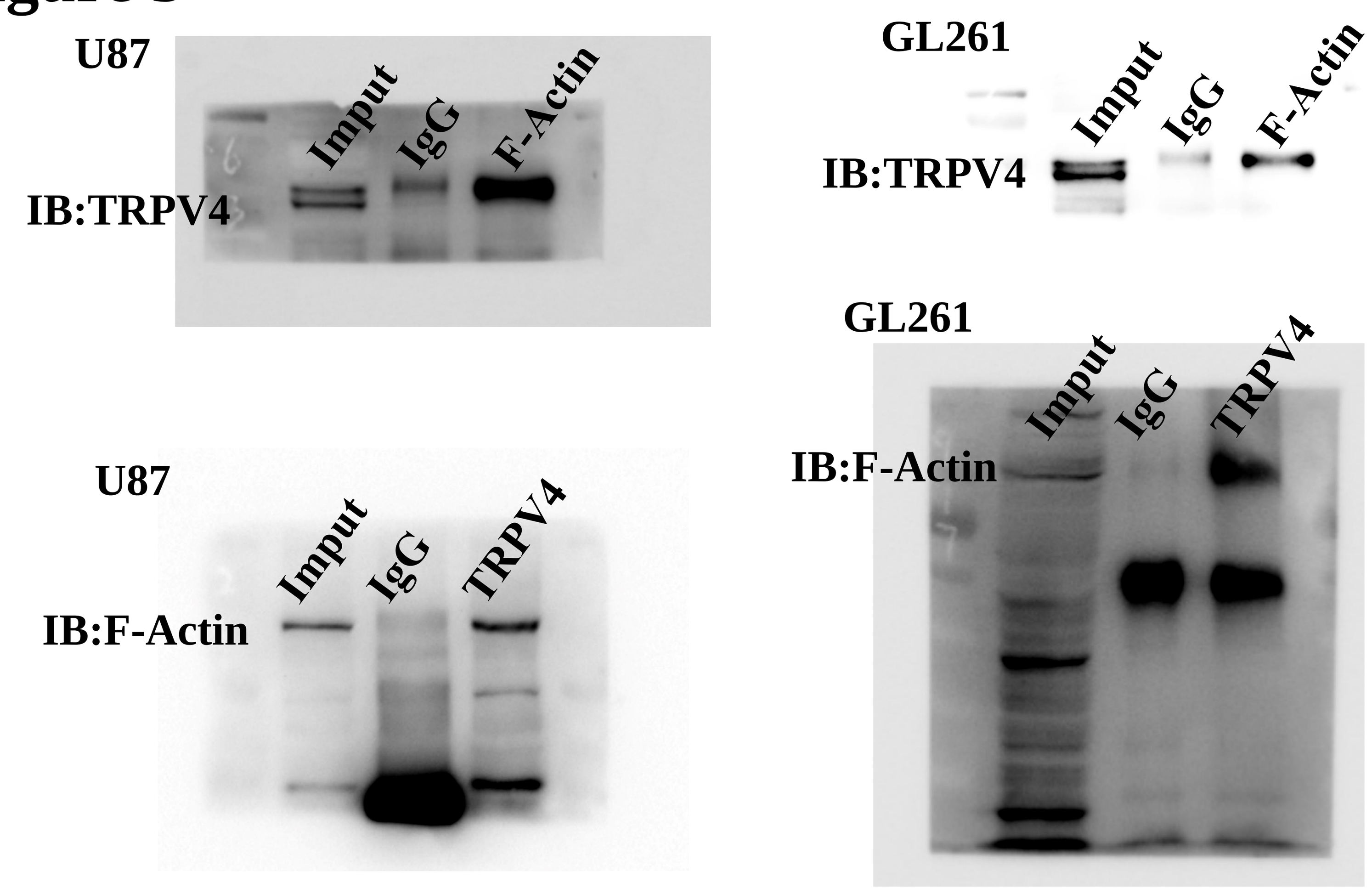

Figure 5

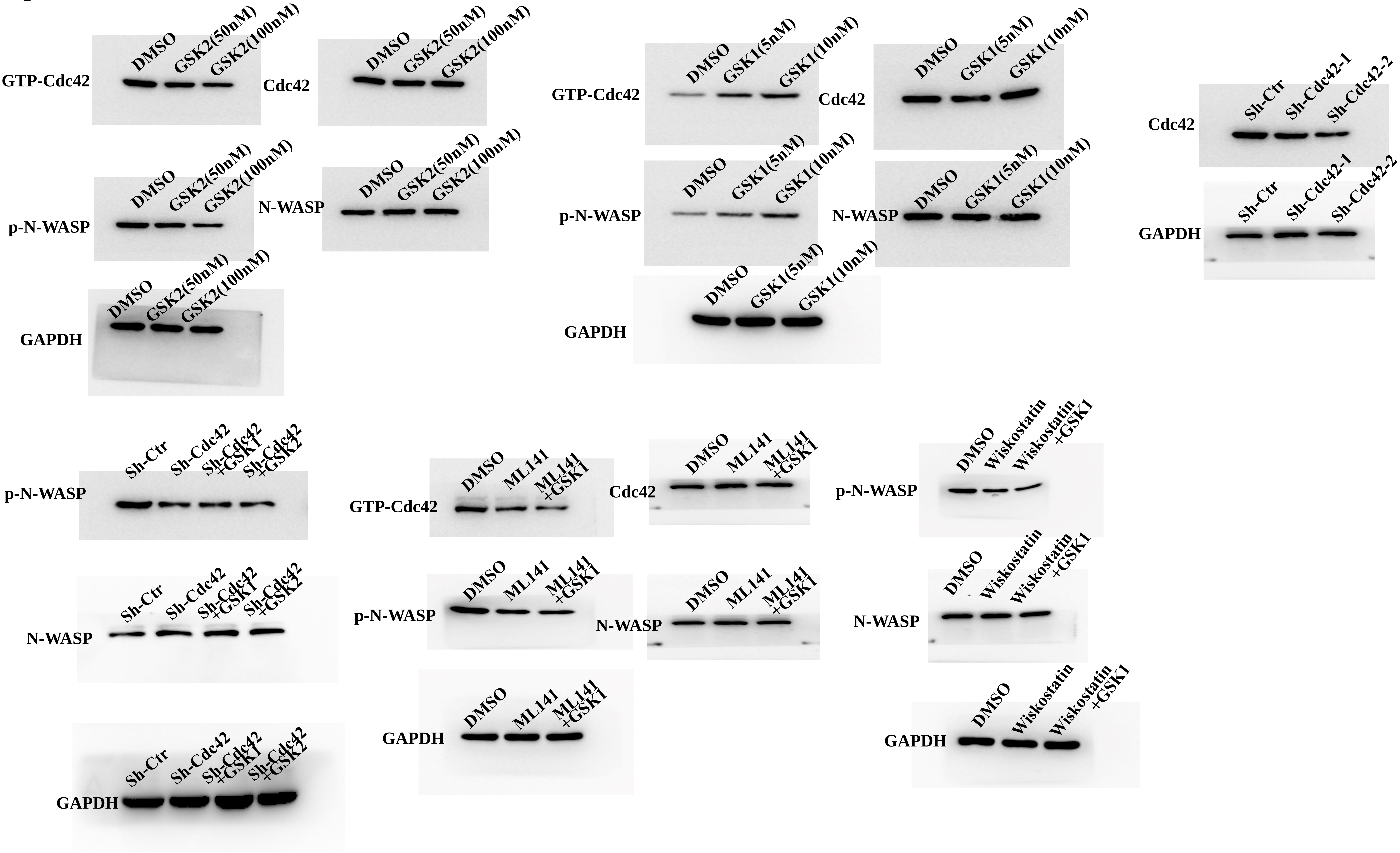

Figure 7

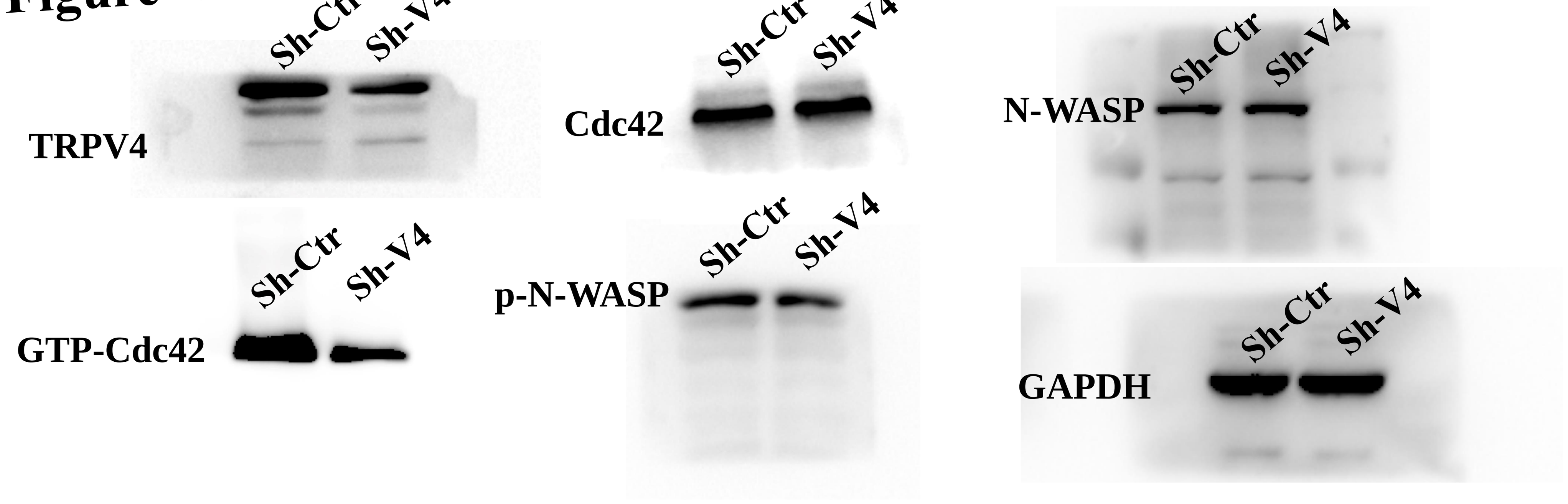

Figure S1

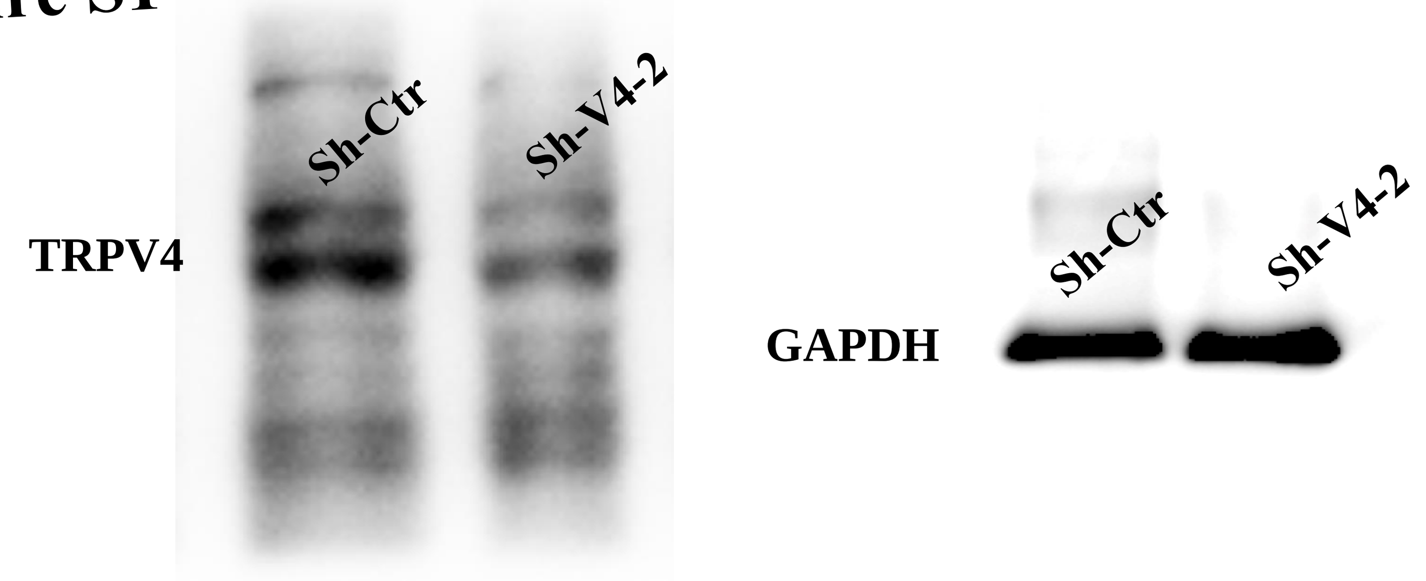

Supplementary Figure 1

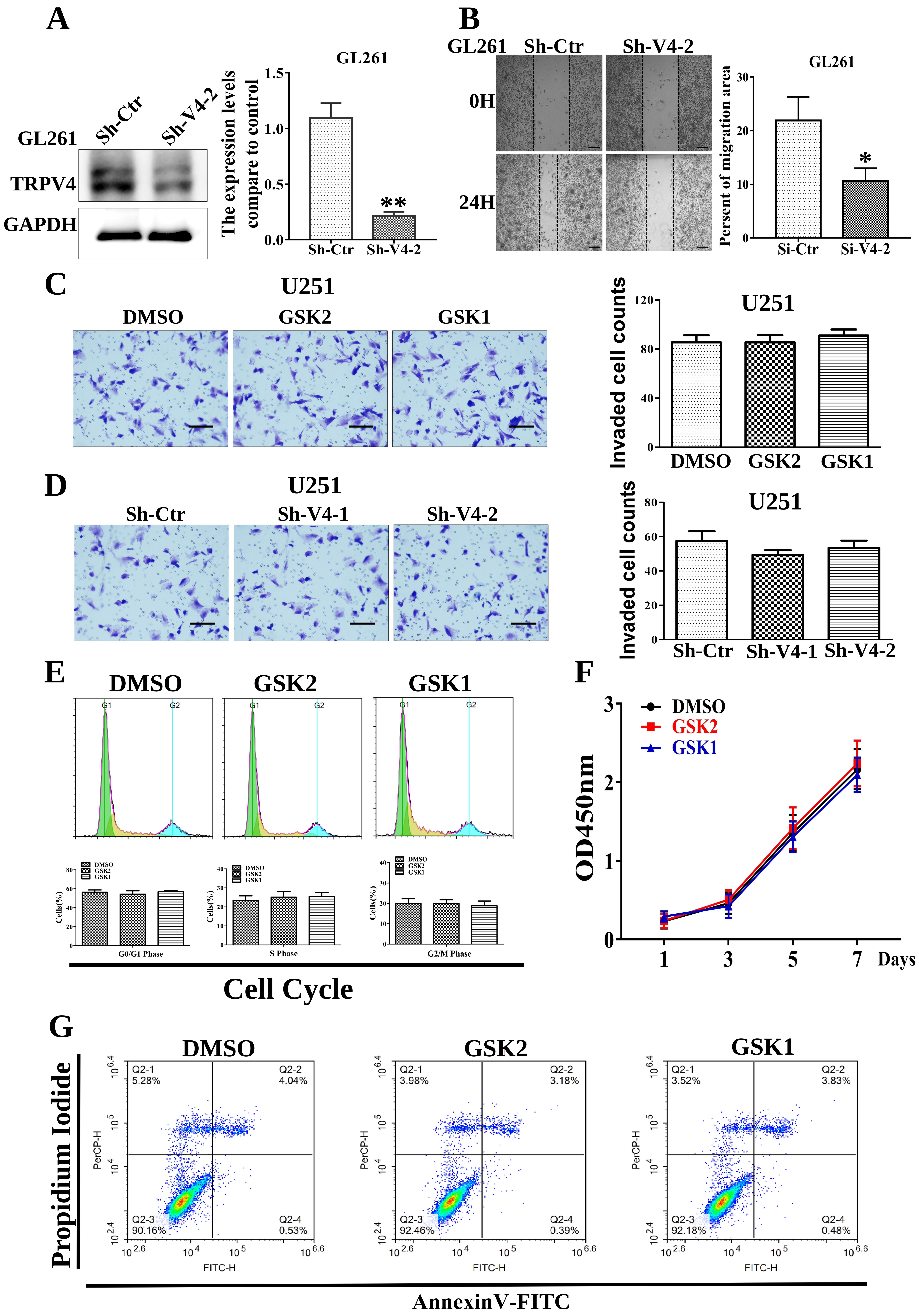

Supplementary Figure 2

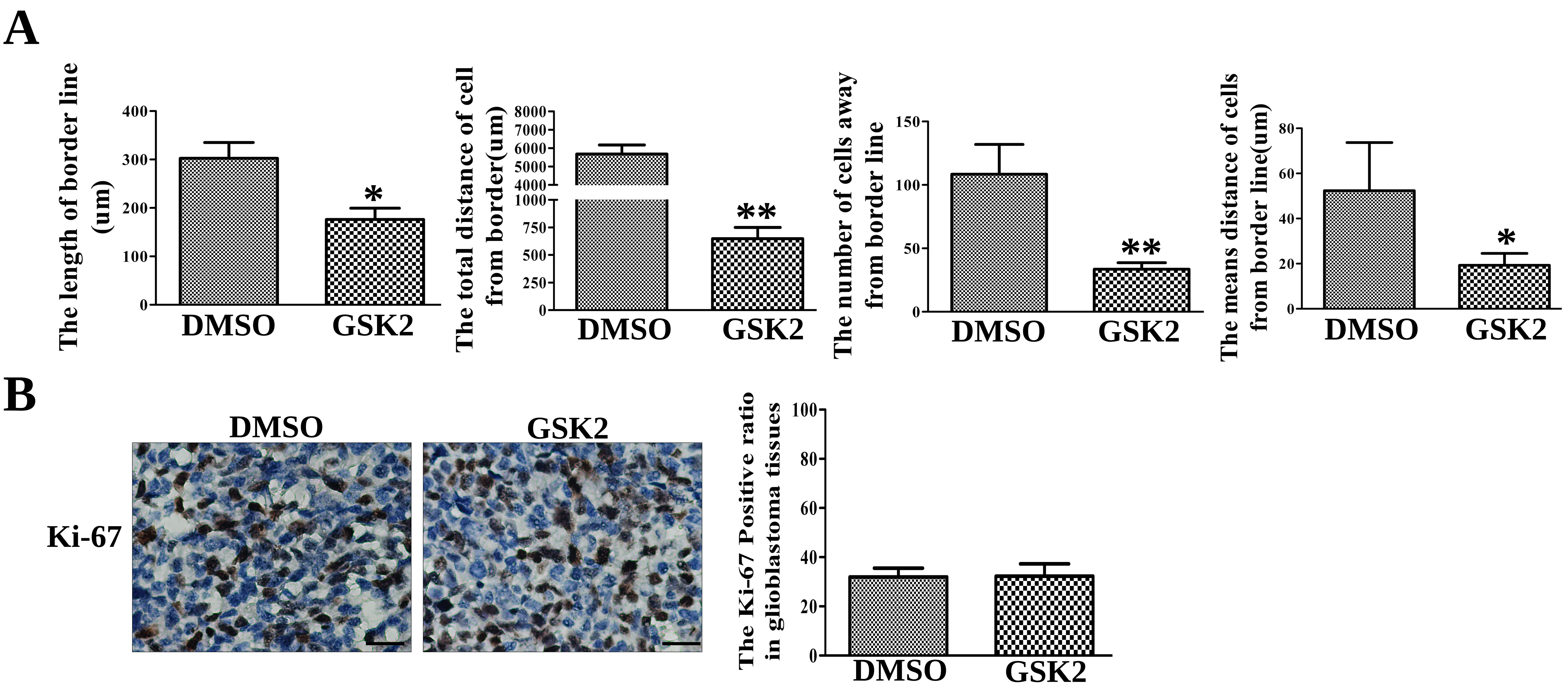

TRPV4 activates the Cdc42/N-wasp pathway to promote glioblastoma invasion by altering cellular protrusions

Wei Yang [1#], Peng-fei Wu [2#], Jian-xing Ma[2], Mao-jun Liao [2], Lun-shan Xu[2,*], and Liang Yi [2,*]

Supplementary Fig. 1: (A-B) The downregulation effect of shRNA targeting TRPV4 (sh-TRPV4-2) was detected by western blot assays and the migration ability after TRPV4 downregulation was detected by wound-healing assay in GL261 cells, scale bar=100 μm. (C) Representative results of Transwell assays of U251 cells treated with TRPV4 agonist and antagonist, scale bar=50 μm. (D) Transwell assays to investigate the invasion ability of U251 when treated with two shRNAs against TRPV4, scale bar=50 μm. (E) Flow cytometry assays to analyze the effect of TRPV4 on the U87 cell cycle by treatment with agonist and antagonist. (F) CCK-8 assays to analyze the effect of TRPV4 on the U87 cell cycle by treatment with agonist and antagonist. (G) Flow cytometry assays to analyze the effect of TRPV4 on U87 cell death when treated with agonist or antagonist. *p<0.05, **P<0.01.

Supplementary Fig. 2: (A)The length of the tumor border line, the number of tumor cells far away from the border line, and the total and mean distance of tumor cells from border line were analyzed to show the invasion states. (B) IHC with Ki-67 antibody to detect the proliferation of transplanted glioblastoma tissues in the indicated groups, scale bar=50 μm. **, p<0.01.
